# Sleep and mental health during the COVID-19 pandemic: findings from an online questionnaire survey in China

**DOI:** 10.3389/fneur.2024.1396673

**Published:** 2024-06-17

**Authors:** Xuqian Liu, Mingyue Liu, Guangyuan Ai, Naijun Hu, Wenhan Liu, Chao Lai, Feng Xu, Zhaohong Xie

**Affiliations:** ^1^Department of Neurology, The Second Hospital, Cheeloo College of Medicine, Shandong University, Jinan, China; ^2^School of Management, Shandong University, Jinan, China

**Keywords:** sleep and mental health, sleep disorder, sleep quality, insomnia, COVID-19, depression, anxiety

## Abstract

**Introduction:**

The online study investigated the sleep, psychological conditions, and risk factors during the wave of transmission of COVID-19 since December 7, 2022.

**Methods:**

We distributed questionnaires through networking mediums to residents to gather information about COVID-19 infection, sleep, and mental status.

**Results:**

During the extraordinary period in China, 91.9% of 1094 participants claimed to be infected with COVID-19, 36.8% reported poor sleep quality, 75.9% reported anxiety, and 65.5% reported depression. In retrospect, people have experienced lower sleep quality, longer sleep latency, enhanced rising time, and decreased sleep efficiency after the infection wave. After adjusting confounding factors, the elderly, women, urban residents, people with comorbidity, anxiety, depression, stress state, and COVID-19 infection have high risks for sleep disorders during the period.

**Discussion:**

The survey indicates that sleep disturbance caused by COVID-19 involves multiple dimensions, such as physiology, psychology, and society. The COVID-19 infection-related sleep problem should be taken seriously. Apart from conventional treatment, psychological issues of insomnia can not be ignored.

## 1 Introduction

The World Health Organization (WHO) declared the coronavirus disease 2019 (COVID-19) a pandemic due to its rapid global spread since 11 March 2020 (1). COVID-19 has spread around the world and internationally, bringing disastrous effects on public mental and physical health, for which severe acute respiratory syndrome coronavirus 2 (SARS-CoV-2) has been identified as the causative agent (2–4). Numerous studies have shown that COVID-19 is highly related to psychological and sleep disturbances, including depression, anxiety, stress, and insomnia. Sleep disruption was expected to occur in the general population during the COVID-19 outbreak (5). In a 6-month cohort study in China, patients confirmed with COVID-19 and discharged from the hospital had symptoms of sleep difficulties (26%) and depression/anxiety (26%) (6). A Greek study has yielded valuable insights into the effect of SARS-CoV-2 infection on mental health and sleep. After 1–2 months of hospital discharge, it was found that depression, anxiety, traumatic stress, and insomnia occupied a total of 143 patients, accounting for 39, 19, 27, and 33%, respectively (7). In an Italian study, 57.1% of 2,291 people were regarded as having poor sleep quality, followed by high anxiety, deep distress, and post-traumatic symptoms of stress in 32.1, 41.8, and 7.6% of people, respectively (8). During the COVID-19 period in Turkey, a study revealed that clinical insomnia was prevalent among 15.9% of 747 university students, and 74% reported poor sleep quality (9). Among 1,171 respondents in China, 46.6% showed symptoms of depression, and 33.0% complained of anxiety (2). Reportedly, it was considered that declining sleep quality was induced by restriction, isolation, quarantine, financial losses, anxiety, stress, and other psychosocial factors (5, 10). It is worth noting that insomnia mediates the influence of perceived stress on depression and anxiety symptoms at the peak of the COVID-19 pandemic (2). To the best of our knowledge, there has been limited research on the physiological effects of COVID-19 infection on insomnia.

A systematic review and meta-analysis concluded that people under mental pressure from large-scale infectious disease outbreaks, including SARS, H1N1, and COVID-19, suffer severe psychological problems, such as anxiety, depression, and insomnia (11). The incidence of psychological problems was higher during the pandemic period of COVID-19 than that of SARS or other epidemic types, owing to the more contiguous and widely spreading virus. Several publications from different countries reported a rising prevalence of sleep disorders in 2020 and examined the effect of the COVID-19 infection on sleep (10). It has been proven that people are confronted with a lack of sleep during the COVID-19 pandemic. Undeniably, there is a close association between sleep and COVID-19.

Several studies have published that insufficient sleep, poor sleep quality, and insomnia are tightly connected with physical and mental disorders (12). Inadequate sleep damages executive functioning, academic performance, and learning capacity, as well as an increased prevalence of affective disorders (13, 14). Therefore, COVID-19-associated sleep disorder is an imperative and non-negligible problem for public health in this global wave of infectious diseases.

China’s policies of epidemic prevention have undergone significant changes since 7 December 2022. Strict quarantine measures and home confinement were no longer taken. Thus, the country faced unprecedented infections with the Omicron variant, which is more contagious and weakly pathogenic than the Delta variant (15). The study aimed to investigate the prevalence of sleep disorders and the risk factors during the high-speed transmission period of COVID-19.

## 2 Methods

### 2.1 Methods of data collection

We conducted the online cross-sectional study in the crowd from 10 January to 9 February 2023. The platform ‘Wenjuanxing’ was chosen for the data collection, and then the designed questionnaire was published and spread mainly throughout the communication application WeChat. We collected data on whether the respondent is infected with COVID-19, sleep quality before and after the COVID-19 pandemic by using the Pittsburgh sleep quality index (PSQI), the degree of drowsiness by Epworth sleepiness scale (ESS), stress by a single-item measure of stress symptoms, depression and anxiety by Health Questionnaire for Depression and Anxiety-4 (PHQ-4), and demographic characteristics (age, sex, region, education, comorbidity, and cigarette smoking, etc.). The study was approved by the ethics committee of the Second Hospital of Shandong University, and informed consent was acquired from all volunteers.

### 2.2 Assessment of sleep and other mental states

The PSQI is one of the most rigorously validated and extensively utilized scales for assessing sleep disorders. It is a self-evaluated questionnaire about sleep quality in 1 month with 18 questions, including 7 dimensions: sleep quality, sleep duration, sleep latency, sleep disturbance, sleep efficiency, use of hypnotics, and daytime dysfunction. Questions are answered and rated on a Likert scale of 0–4, with a total score ranging from 0 to 28. The higher the total score, the worse the sleep quality (Cronbach’s *α* = 0.83, McDonald’s *ω* = 0.81) (9, 16). The cutoff score is 8 (17–19), indicating poor sleep quality according to Sleep Medicine-Second Edition (20).

The Insomnia Severity Index (ISI) was used to determine the severity of clinical insomnia. The 7-item self-reported scale rated on a scale of 0–4 scores ranges from 0 to 28 (Cronbach’s *α* = 0.81, McDonald’s *ω* = 0.85) (9, 21). The degree of insomnia is categorized into four formal levels: no clinical significance (0–7), subclinical (8–14), moderate (15–21), and severe (22–28) insomnia (20). Hence, a score of 8 or above is regarded as insomnia above the clinical threshold in this study (20, 22).

The Patient Health Questionnaire-4 (PHQ-4) is a brief screening scale for identifying potential populations with depression and anxiety, constructed by integrating the Patient Health Questionnaire-2 (PHQ-2) and the Generalized Anxiety Disorder-2 scale (GAD-2). The screener consists of two major dimensions: depression and anxiety (23).

It also assesses mental health, social and general health, pain, and physical functioning impairment (Cronbach’s α > 0.80) (23). The depression subscale, which includes two items from the PHQ-2, is one of the Patient Health Questionnaire-9 (PHQ-9) score criteria for depressive disorders. The two-question scale is scored from 0 to 3 (ranked as ‘not at all, several days, more than half the days, and nearly every day’) and has a cut point of 3 with a sensitivity of 83% and specificity of 90% for the diagnosis of depression (24). Similarly, the severity of anxiety is assessed by GAD-2, derived from the Generalized Anxiety Disorder-7 scale, as a score of 3 or above, with satisfied sensitivity (88%) and specificity (81–83%) (25).

The cognition level of participants was surveyed with a simplified 4-question scale, self-evaluated from 0 to 3 points (never, a little, moderate, and definite) to quantify the degree of cognition decline, by which high total scores indicate lower cognitive capacity (Cronbach’s *α* = 0.87) (26, 27).

The severity of mental pressure incorporated items measuring sleep disturbance and wellbeing, with higher scores indicating more severe stress symptoms by a 5-level Likert scale (Cronbach’s *α* = 0.88–0.90) (28, 29).

Post-traumatic stress disorder (PTSD). An abbreviated screening scale was used to quantify PTSD symptoms on a 1–5 scale, with a cutoff score of 4 deferred as current PTSD in the past month (30). The two-item version deprived of the PTSD Checklist is well constructed, with a sensitivity of 97% and specificity of 65% (Cronbach’s *α* = 0.80–0.90) (31, 32).

The Epworth Sleeping Scale (ESS) is an 8-item validated questionnaire that assesses the likelihood of falling asleep during a variety of daily living situations, which is clinically defined as excessive daytime sleepiness with a total score of >10 (Cronbach’s *α* = 0.81) (33).

### 2.3 Methods for data manipulation and analysis

The continuous variables were represented with median and interquartile ranges, and the categorical variables were described with the number of cases and percentages. For the analysis, volunteers were defined as “participants with poor sleep quality” and “participants with normal sleep quality” based on sleep quality. And besides, they were also divided into group “participants with insomnia” and “participants without insomnia” according to the criteria for insomnia. Wilcoxon rank-sum test and chi-square were performed to find out the significance. Linear logistic regression was used to adjust all confounders. A multivariate regression model has been implemented to evaluate factors associated with poor sleep quality in the sample, including all independent variables. A *p*-value less than 0.05 for the statistical test was considered significant. All statistical analyses were conducted with SPSS 26.

## 3 Results

The invalid questionnaires were rejected from 1,187 complete questionnaires, of which 1,094 participants’ data were finally obtained for statistical analysis. The information from a total of 1,094 respondents is presented and analyzed in Tables 1–4. The age of participants ranged from 15 to 85, with a median of 31 (IQR: 36, 43) years. Males accounted for 30% (*n* = 306) and females accounted for 70% of participants (*n* = 788). Most of the respondents (89%) were from the city, with the rest (11%) living in rural areas. People with bachelor’s degrees or above (78.6%) dominated the online questionnaire survey. A total of 23.3% of the participants reported single or more comorbidities.

**Table 1:** Risk factors for poor sleep quality among respondents during the COVID-19 pandemic (*N* = 1,094).

Factor	Total sample	Poor sleep quality	Normal sleep quality	*p-*value
Total (*n*, %)	1,094 (100)	691 (63.2)	403 (36.8)	–
Gender (*n*, %)				
Male	306 (30.0)	161 (23.3)	145 (36.0)	**0.001**
Female	788 (70.0)	530 (76.7)	258 (64.0)	
Occupation (*n*, %)				**0.001**^**a**^
Labor worker	147 (13.0)	96 (13.9)	51 (12.7)	
Mental worker	513 (46.9)	342 (49.5)	171 (42.4)	**<0.05**
Student	122 (11.2)	53 (7.7)	69 (17.1)	**<0.05**
Other	312 (28.5)	200 (28.9)	112 (27.8)	>0.05
Residence (*n*, %)				
Urban	974 (89.0)	629 (91)	345 (85.6)	**0.006**
Rural	120 (11.0)	62 (9)	58 (14.4)	
Education level (*n*, %)				0.054
Junior high school and below	57 (5.21)	45 (6.5)	34 (8.4)	
Technical College	155 (14.1)	87 (12.6)	68 (16.9)	
Bachelor’s degree or above	860 (78.6)	559 (80.9)	301 (74.7)	
Comorbidity (*n*, %)				
Yes	255 (23.3)	177 (25.6)	78 (19.4)	**0.018**
No	839 (76.7)	514 (74.4)	325 (80.6)	
Smoking (*n*, %)				0.529
Never	933 (85.3)	596 (86.3)	337 (83.6)	
Sometimes	68 (6.2)	39 (5.6)	29 (7.2)	
Ever	27 (2.5)	18 (2.6)	9 (2.2)	
Present	66 (6.0)	38 (5.5)	28 (6.9)	
Infected with COVID-19 (*n*, %)				
No	39 (3.6)	9 (1.3)	30 (7.4)	
Experiencing	21 (1.9)	16 (2.3)	5 (1.2)	**<0.05**
Having experienced	1,005 (91.9)	654 (94.6)	351 (87.1)	**>0.05**
Unclear	29 (2.6)	12 (1.7)	17 (4.2)	>0.05
Symptom (*n*, %)				**0.001**
Yes	866 (80.0)	615 (89)	251 (62.3)	
No	228 (20.0)	76 (11)	152 (37.7)	
Anxiety (*n*, %)				
Yes	830 (75.9)	566 (81.9)	264 (65.5)	**0.001**
No	264 (24.1)	125 (18.1)	139 (34.5)	
Depression (*n*, %)				
Yes	717 (65.5)	567 (82.1)	150 (37.2)	**0.001**
No	377 (34.5)	124 (17.9)	253 (62.8)	
PTSD (*n*, %)				
Yes	412 (37.7)	329 (47.6)	83 (20.6)	**0.001**
No	682 (62.3)	362 (52.4)	320 (79.4)	
Daytime sleepiness (*n*, %)				
Yes	809 (73.9)	565 (69.8)	244 (30.2)	**0.001**
No	285 (26.1)	126 (44.2)	159 (55.8)	

Qualitative data were analyzed by chi-square test. Poor sleep quality (PSQI > = 8). Normal sleep quality (PSQI <8). Bolding *p*-value showed significant differences (*p* < 0.05). ^a^Multiple comparisons by Bonferroni test.

**Table 2:** Risk factors for poor sleep quality among respondents during the COVID-19 pandemic (*N* = 1,094).

Factor	Total sample	Poor sleep quality	Normal sleep quality	*p-*value
Total (*n*, %)	1,094 (100)	691 (63.2)	403 (36.8)	–
Age M (IQR)	31.0 (36.0, 43.0)	32.0 (37.0, 43.0)	28.0 (35.0, 43.0)	**0.003**
BMI M (IQR)	20.4 (22.2, 24.2)	20.5 (22.2, 24. 1)	20.2 (22.2, 24.2)	0.883
Duration of infection with COVID-19 M (IQR)	6.0 (7.0, 10.0)	7.0 (8.0, 10.0)	5.0 (7.0, 8.0)	**0.001**
Frequency of vaccination with COVID-19 M (IQR)	3.0 (3.0, 0)	3.0 (3.0, 3.0)	3.0 (3.0, 3.0)	0.186
Degree of stress M (IQR)	1.0 (2.0, 3.0)	2.0 (2.0, 4.0)	1.0 (1.0, 2.0)	**0.001**
Cognitive level M (IQR)	0 (3.0, 5.0)	2.0 (4.0, 6.0)	0 (0, 3.0)	**0.001**

Quantitative data were analyzed using the Wilcoxon rank-sum test. Poor sleep quality (PSQI > = 8); Normal sleep quality (PSQI <8). Bolding *p*-value showed significant differences (*p* < 0.05).

**Table 3:** Risk factors for insomnia among respondents during the COVID-19 pandemic (*N* = 1,094).

Factor	Total sample	Insomnia	Normal sleep	*p*-value
Total (*n*, %)	1,094 (100)	981 (89.7)	113 (10.3)	–
Gender (*n*, %)				
Male	306 (30.0)	268 (27.3)	38 (33.6)	0.157
Female	788 (70.0)	713 (72.7)	75 (66.4)	
Occupation (*n*, %)				0.280
Labor worker	147 (13.0)	127 (12.9)	20 (17.7)	
Mental worker	513 (46.9)	469 (47.8)	44 (38.9)	
Student	122 (11.2)	108 (11.0)	14 (12.4)	
Other	312 (28.5)	227 (28.2)	35 (31.0)	
Residence (*n*, %)				
Urban	974 (89.0)	879 (89.6)	95 (84.1)	0.075
Rural	120 (11.0)	102 (10.4)	18 (15.9)	
Education (*n*, %)				0.785
Junior high school and below	57 (5.21)	69 (7.0)	10 (8.8)	
Technical College	155 (14.2)	139 (14.2)	16 (14.2)	
Bachelor’s degree or above	860 (78.6)	773 (78.8)	87 (77.0)	
Comorbidity (*n*, %)				
Yes	255 (23.3)	236 (24.1)	19 (16.8)	0.085
No	839 (76.7)	745 (75.9)	94 (83.2)	
Smoking (*n*, %)				0.615
Never	933 (85.3)	840 (85.6)	93 (82.3)	
Sometimes	68 (6.2)	61 (6.2)	7 (6.2)	
No	285 (26.1)	215 (21.9)	70 (61.9)	
Ever	27 (2.5)	24 (2.4)	3 (2.7)	
Present	66 (6.0)	56 (5.7)	10 (8.8)	
Infected with COVID-19 (*n*, %)				**0.005**^**a**^
No	39 (3.6)	28 (2.9)	11 (9.7)	
Experiencing	21 (1.9)	20 (2.0)	1 (0.9)	**<0.05**
Having experienced	1,005 (91.9)	907 (92.5)	98 (86.7)	>0.05
Unclear	29 (2.6)	26 (2.7)	3 (2.7)	**<0.05**
Symptom (*n*, %)				**0.001**
Yes	866 (80.0)	805 (82.1)	61 (54.0)	
No	228 (20.0)	176 (17.9)	52 (46.0)	
Anxiety (*n*, %)				
Yes	830 (75.9)	687 (70.0)	18 (15.9)	**0.001**
No	264 (24.1)	294 (30.0)	95 (84.1)	
Depression (*n*, %)				
Yes	717 (65.5)	697 (71)	20 (17.7)	**0.001**
No	377 (34.5)	284 (29)	93 (82.3)	
PTSD (*n*, %)				
Yes	412 (37.7)	399 (40.7)	13 (11.5)	**0.001**
No	682 (62.3)	582 (59.3)	100 (88.5)	
Daytime sleepiness (*n*, %)				
Yes	809 (73.9)	766 (78.1)	43 (38.1)	**0.001**
No	285 (26.1)	215 (21.9)	70 (61.9)	

Qualitative data were analyzed by chi-square test. Insomnia (ISI > = 8). Normal sleep (ISI < 8). Bolding *p*-value showed significant differences (*p* < 0.05). ^a^Multiple comparisons by Bonferroni test.

**Table 4:** Risk factors for insomnia among respondents during the COVID-19 pandemic (*N* = 1,094).

Factor	Total sample	Insomnia	Normal sleep	*p-*value
Total (*n*, %)	1,094 (100)	981 (89.7)	113 (10.3)	–
Age M (IQR)	31.0 (36.0, 43.0)	36.0 (30.0, 43.0)	36.0 (32.0, 44.0)	0.655
BMI M (IQR)	20.4 (22.2, 24.2)	22.2 (20.4, 24.2)	22.2 (20.3, 24.6)	0.416
Duration of infection with COVID-19 M (IQR)	6.0 (7.0, 10.0)	7.0 (6.0, 10.0)	7.0 (5.0, 7.0)	**0.001**
Frequency of vaccination with COVID-19 M (IQR)	3.0 (3.0, 0)	3.0 (3.0, 3.0)	3.0 (3.0, 3.0)	0.731
Degree of stress M (IQR)	1.0 (2.0, 3.0)	2.0 (1.0, 3.0)	1.0 (1.0, 1.0)	**0.001**
Cognitive level M (IQR)	0 (3.0, 5.0)	4.0 (1.0, 5.0)	0 (0, 0)	**0.001**

Quantitative data were analyzed by the Wilcoxon rank-sum test. Insomnia (ISI > = 8). Normal sleep (ISI < 8). Bolding *p*-value showed significant differences (*p* < 0.05).

**Figure 1:**
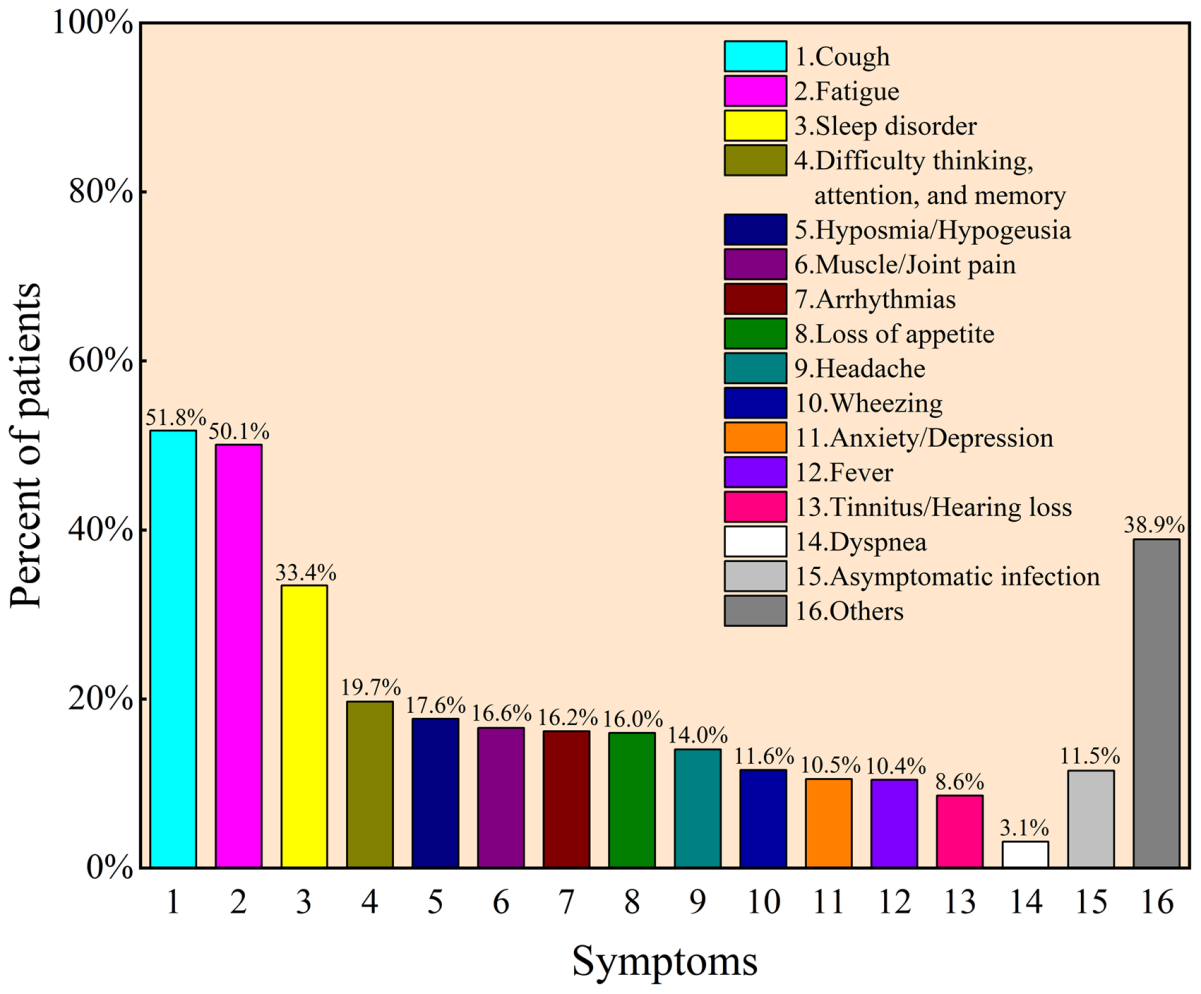
Frequency of post-COVID-19 syndrome after initial diagnosis as reported by participants for COVID-19 (*N* = 1,026).

Of the total 1,094 participants, 91.9% (*n* = 1,005) have been infected with COVID-19, 1.9% (*n* = 21) claimed to be experiencing infection, and 3.6% (*n* = 39) clearly stated that they have not been infected, leaving 2.6% unclear. Among participants infected with COVID-19, 88.4% (*n* = 908) complained of at least one symptom. The most common symptom is cough (531, 51.8%), followed by fatigue (514, 50.1%), sleep disorder (343, 33.4%), and difficulty thinking/attention/memory (202, 19.7%) (Figure 1). The median infection duration was 6 days (IQR: 7, 10).

It has been found that 36.8% of the participants (*n* = 691) reported having lower PSQI scores, indicating poor sleep quality after the emergence of the COVID-19 pandemic (Tables 1, 2). Age, gender, BMI, occupation, habitation, and comorbidity were closely associated with sleep quality. Females (compared with males, OR = 1.84, 95%CI: 1.41–2.42), urban settings (compared with rural, OR = 1.71, 95%CI: 1.17–2.50), and comorbidities (compared with no comorbidities, OR = 1.4, 95%CI: 1.06–1.94) had significantly higher percentages of poor sleep quality. The group with poor sleep quality was 2 years younger (95%CI: −3– −1) than the group with ordinary sleep quality. Although smoking and education level showed a marginally significant association with sleep quality, we did not detect a statistical interaction of sleep quality with BMI and occupation.

Importantly, we discovered that COVID-19 infection, the duration of infection, and related symptoms were significantly associated with sleep quality, except that the frequency of COVID-19 vaccine administrations showed no discernible influence on sleep. More specifically, participants experiencing COVID-19 infection exhibited a higher risk of poor quality compared to the uninfected or already infected population. However, there was no significant association between uninfected and already infected groups. The median infection duration was 7.0 (IQR: 8.0, 10.0) for the poor sleep quality group and 5.0 (IQR: 7.0, 8.0) for the poor sleep quality group, respectively. The median difference between the two groups was 2 (poor relative to normal, 95%CI: 1.0–2.0), which demonstrated that a long period of infection by COVID-19 was likely to cause decreasing sleep quality. Respondents bearing one or more infection symptoms induced by COVID-19 had a 3.9 higher risk for abnormal sleep quality (OR = 4.9, 95%CI: 3.59–6.70). The times of vaccination did not show a significant difference in participants with poor sleep quality compared with the ordinary group.

The associations between the epidemiological character of COVID-19 infection and poor sleep quality risk were similar to those with insomnia. Correspondingly, COVID-19 infection, long duration of infection, and existing related symptoms were significantly associated with sleep disorders, and frequency of vaccination showed no connection with insomnia (Tables 3, 4). Nevertheless, no statistically significant associations were observed between insomnia risk and demographic variables such as age, gender, BMI, habitation, comorbidities, smoking status, or educational attainment.

Similarly, mental state, for instance, stress, anxiety, depression, PTSD, and cognitive level, had a statistically significant impact on both sleep quality and insomnia. Besides, excessive daytime sleep plays an essential role in sleep disturbance. It has been observed that respondents who were assessed as having anxiety, depression, PTSD, or ESS had a 7.6 (OR = 8.6, 95%CI: 6.49–11.41), 6.1 (OR = 7.1, 95%CI: 5.83–10.21), 2.5 (OR = 3.5, 95%CI: 2.64–4.65), and 1.9 (OR = 2.9, 95%CI: 2.21–3.86) times higher chance, respectively, of developing poor sleep quality than respondents who did not. It has also been detected that anxiety, depression, PTSD, or ESS had 12.3 (OR = 12.3, 95%CI: 7.32–20.79), 11.4 (OR = 11.4, 95%CI: 6.91–18.86), 5.3 (OR = 5.3, 95%CI: 2.92–9.53), and 5.8 (OR = 5.8, 95%CI: 3.85–8.73) times of risk for insomnia. The median cognition scores are 2.0 (IQR: 4.0, 6.0) for participants with poor sleep quality and 0 (IQR: 0, 3.0) among participants in normal sleep conditions, with a significant score difference of 3.0 (95%CI: 2.0–3.0), which is also observed in the insomnia analysis.

There are noticeable changes in total sleep quality and sleep parameters of the population before and after the highest transmission period (Table 5). The median number of PSQI and sleep latency are apparently enhanced from 5 (IQR: 3.0, 8.0) to 6.0 (IQR: 3.0, 9.0) and from 20.0 (IQR: 10.0, 30.0) to 30.0 (IQR: 10.0, 40.0), respectively. Respondents reported lower sleep quality and longer sleep latency since the pandemic peak. There are slightly advanced rise time (MD = 0, 95%CI: 0-0, *p* = 0.026) and diminished sleep efficiency (MD = 0, 95%CI: 0-0, *p* = 0.023) compared to previous period. The paired Kruskal–Wallis test revealed that respondents were more likely to wake up earlier in the morning than before. Besides, it was reported that the peak period may lead to extended sleep latency. We found no significant association between sleep duration and widespread COVID-19.

**Table 5:** Differences in sleep quality and sleep parameters between before and after the pandemic by the paired rank sum test method.

	Before pandemic	After pandemic	*Z*-value	*p*-value	MD^e^ 95%CI
PSQI^a^	5.0 (3.0, 8.0)	6.0 (3.0, 9.0)	−22.12	**0.001**	3.0 (3.0, 3.5)
Sleep duration^b^	7.0 (7.0, 8.0)	6.0 (7.0, 8.0)	−1.46	0.146	0 (0, 0)
Sleep latency^c^	20.0 (10.0, 30.0)	30.0 (10.0, 40.0)	−11.21	**0.001**	2.5 (0, 3.5)
Rising time^b^	6.0 (6.0, 7.0)	6.0 (7.0, 8.0)	−2.23	**0.026**	0 (0, 0)
Sleep efficiency^d^	87.5 (80.0, 100.0)	87.5 (80.0, 100.0)	−2.28	**0.023**	0 (0, 0)

^a^Total score of PSQI. ^b^(hour). ^c^(minute). ^d^(%). ^E^Median difference. Bolding *p*-value showed significant differences (*p* < 0.05).

After adjusting demographic factors, a multivariate linear regression model was performed, and statistical significance was observed in comorbidity, COVID-19 infection, anxiety, and stress (Table 6). Nevertheless, the duration of infection, depression, PTSD, and frequency of vaccination have no significant effect on sleep.

**Table 6:** Multiple linear regression models for participants’ PSQI and ISI scores.

	PQSI				ISI			
	B	*t*	*p*	95%CI	*B*	*t*	*p*	95%CI
Age	0.047	3.749	**0.001**	0.023–0.072	0.036	1.996	**0.046**	0.001–0.071
Female^a^	0.835	2.976	**0.003**	0.284–1.684	0.362	0.909	0.364	−0.420–1.144
Urban^b^	0.973	2.686	**0.007**	0.262–1.147	0.641	1.245	0.213	−0.369–1.651
Comorbidity	0.596	2.119	**0.034**	0.044–1.147	1.571	3.935	**0.001**	0.788–2.354
Depression	0.159	1.179	0.239	−0.105–0.423	0.007	0.036	0.971	−0.368–0.382
Anxiety	0.350	2.550	**0.011**	0.081–0.619	0.716	3.674	**0.001**	0.334–1.099
Stress	1.789	11.939	**0.001**	1.495–2.083	3.453	16.221	**0.001**	3.035–3.871
PTSD	−0.095	−1.238	0.216	−0.247–0.056	0.187	1.713	0.087	−0.027–0.402
Infected with COVID-19								
Experiencing	4.185	4.278	**0.001**	2.266–6.105	3.653	2.628	**0.009**	0.925–6.380
Having experienced	2.926	4.921	**0.001**	1.759–4.093	2.353	2.786	**0.005**	0.696–4.011
Unclear	1.543	1.723	0.085	−0.214–3.300	1.413	1.111	0.267	−1.083–3.909
Duration^c^	0.003	0.774	0.439	−0.004–0.010	0.009	1.738	0.083	−0.001–0.018
Vaccine^d^	−0.187	−1.458	0.145	−0.439–0.065	−0239	−1.309	0.191	−0.597–0.119

PSQI, Pittsburgh Sleep Quality Index. ISI, Insomnia Severity Index. The bold values in the table represent a *p*-value of <0.05, indicating that statistical significance has been achieved. ^a^Compared with males. ^b^Compared with urban. ^c^Duration of COVID-19 infection. ^d^Frequency of coronavirus vaccine.

## 4 Discussion

This is an online questionnaire-based study on the sleep and mental status of residents recruited from 10 January to 9 February 2023, the high-speed transmission period of COVID-19. In our study, 91.9% of 1,094 participants were confirmed SARS-CoV-2 positive, 36.8% complained of poor sleep quality, and 89.7% reported insomnia. The most common symptom is cough (531, 51.8%), followed by fatigue (514, 50.1%) and sleep disorders. Based on a German cohort study of 667 respondents, the most common persistent symptoms were neurological disorders (61.5%), fatigue (57.1%), and sleep disturbance (57.0%) (34). Consistent with the findings, approximately half of the respondents exhibited symptoms of fatigue after contracting COVID-19; specifically, a Turkish study reported 56.4% of 275 participants (35).

Insomnia is the most common sleep disorder, with a reported prevalence of 15% in China, lower than in Western countries (for example, 37.2% in France and Italy, 27.1% in the United States, 25.3% in Greece, 20.8% in Spain, and 50.5% in Poland), but similar to Asian countries (for example, 15.3% in Japan and 17.3% in Singapore) (36). During the COVID-19 period in Turkey, a study revealed that clinical insomnia was prevalent among 15.9% of 747 university students, and 74% reported poor sleep quality (9). A meta-analysis including 948,882 individuals revealed that the occurrence rate of insomnia symptoms varied from 29.7% among the general public to 58.4% among university students during epidemic outbreaks, including COVID-19 (11). It is reported by a Canadian online survey that clinically meaningful sleep difficulties increased enormously from 36% of pre-outbreak to 50.5% during the COVID-19 outbreak between 3 April and 24 June 2020 (37). It was also found that in our survey, the proportion of poor sleep quality increased from 28 to 50.5% before and during the COVID-19 outbreak. People were more likely to report lower sleep quality, longer sleep latency, enhanced rising time, and decreased efficiency after experiencing the infection wave. Specifically, a significant increase in sleep difficulties existed in sleep initiation and early morning awakenings, consistent with the Canadian study. Besides, there was also an incredible reduction in sleep efficiency, which is not mentioned.

We also observed a statistically significant relationship between sleep quality and variables such as age, gender, BMI, occupation, habitation, comorbidity, depression, anxiety, COVID-19 infection, duration of infection, and related symptoms. Additionally, contracting COVID-19 posed a heightened risk of deteriorated sleep quality compared to non-infected individuals. Extended periods of COVID-19 infection were associated with an increased likelihood of developing sleep disturbance.

Demographically, older people were observed to be prone to sleep issues related to COVID-19, which is in line with other studies (38). This may be explained by some hypotheses that the impaired circadian rhythm and secretion of melatonin with aging are more vulnerable to COVID-19 due to the increasing binding of the coronavirus to ACE_2_ receptors (39). Aligned with the formal survey, females are more likely to develop sleep difficulties with an exaggerated response to stressful events, such as pandemics and natural disasters (7, 40). After excluding the impact of demographics, COVID-19 infection, anxiety, depression, and stress are responsible for sleep disturbances after the outbreak of COVID-19. However, no significant difference was observed between vaccination and sleep disorders.

Similar to our research, a meta-analysis in 2022 indicated an increasing prevalence of mental health issues over time among the general public, healthcare workers, and university students during the COVID-19 pandemic (11). In a 6-month cohort study of COVID-19 in discharged patients, 26% of 1,655 reported sleep difficulties, and symptom anxiety or depression accounted for 23% of the population (6), which is significantly lower than what is reported in our study. This may be explained by the fact that our research was conducted soon after the national outbreak, when people tended to have more sleep problems due to anxiety, panic, isolation, and concerns about being infected and deteriorating situations due to the pandemic, while the formal study focused on patients who had been discharged within 6 months. Notably, a web-based study conducted in 2020 revealed that during the initial outbreak of the COVID-19 pandemic in China, depression, anxiety, insomnia, and acute stress symptoms affected 20.4, 27.1, 27.5, and 21.2% of 3,730 individuals over the age of 50, respectively. Correspondingly, our study revealed a marked escalation in the prevalence of anxiety (75.9%), depression (65.0%), and insomnia (89.7%) in 2023. Insomnia occurred in an ascending trend as the virus spread using the same cutoff value in the ISI. According to previous research, the prevalence of insomnia, anxiety, and depression has significantly increased and is associated with risk factors including female sex, comorbidity, mental disorders, COVID infection, infection of colleagues or family members, close contact with infected individuals, increased attention to the epidemic, and quarantine experience (11). The bi-directional relationship between sleep and psychological issues may explain the fact that overloaded psychological reactions to the wave of the pandemic worsen sleep, and sleep may worsen stress, anxiety, and depression conversely (40). Prolonged sleep onset latency may lead to high arousal before sleep and concerns, which is, in turn, connected with the occurrence of anxiety (39). Similarly, the overall target population with poor sleep conditions has higher rates of anxiety-lengthened sleep latency.

Fatigue and depression often occur as concomitants, with a striking overlap in COVID-19 symptom constellations (41). In addition to central, peripheral, and psychological changes, post-infection fatigue may also mediate the relationship between COVID-19 infection and psychiatric symptoms, as suggested by a foreign survey (35). Stress involving lockdowns and atypical schedules may make people more vulnerable to sleep disturbances. Considering the high prevalence of anxiety, depression, fatigue, and stress symptoms, it is difficult to separate the direct influence of the virus on physical health from the effect of an epidemic on the population (42).

Coherently, the public has shown an elevated rate of insomnia, as reported in previous research (11, 39, 43). Similarly, the diagnosis of anxiety and insomnia was more prevalent after the COVID-19 pandemic in the present study (37), which aligns with the investigation of psychological symptoms during the SARS and MERS outbreaks (42). According to Rogers et al., the etiology of COVID-related mental symptoms is multi-dimensional, including the direct effects of viral infection, hyperinflammatory state, cerebrovascular events caused in part by a procoagulant state, medical interventions, social segregation, the psychological effects of severe illness, the risk of contagion, concerns about infecting others, and defamation (42). Rarely, SARS-CoV-2-related psychiatric symptoms have been related to encephalitis and hypoxia in the brain. The survey suggests that COVID-19 infection leads to sleep disorders in multiple dimensions involving physiology, psychology, and society. The COVID-19 infection-related sleep problem should be valued. Apart from conventional treatment, psychological issues cannot be ignored for insomnia. Our research provides a reference for the future spread of infectious diseases.

There are several limitations to our study. First, inaccurate data were obtained due to subjectivity and impatience throughout the online questionnaire method, and sleep and overall mental conditions were explored without a definite diagnosis. Second, the prevalence of infection with COVID-19 was so high in the sample that a negative comparison group was lacking, leading to selection bias. However, it is inevitable due to the high transmission rate of COVID-19. Besides, the sampling population is not well representative and is limited by the distribution of questionnaires, which were mainly forwarded around the network of researchers around them. More rigorous data collection methods and biomarkers such as neutrophils, C-reactive protein, ferritin, and interleukin-6 should also be valued during the following study.

## Data availability statement

The data analyzed in this study is subject to the following licenses/restrictions: academic communication. Requests to access these datasets should be directed to xie_zhaohong@sdu.edu.cn.

## Ethics statement

The studies involving humans were approved by Scientific Research Ethics Committee of the Second Hospital of Shandong University. The studies were conducted in accordance with the local legislation and institutional requirements. Written informed consent for participation in this study was provided by the participants’ legal guardians/next of kin.

## Author contributions

XL: Data curation, Formal analysis, Writing – original draft, Writing – review & editing. ML: Data curation, Resources, Writing – review & editing. GA: Data curation, Resources, Software, Writing – review & editing. NH: Data curation, Formal analysis, Resources, Writing – review & editing. WL: Resources, Visualization, Writing – review & editing. CL: Methodology, Project administration, Resources, Supervision, Writing – review & editing. FX: Investigation, Methodology, Resources, Writing – review & editing. ZX: Funding acquisition, Investigation, Methodology, Writing – review & editing.
